# Exploring a Co-Designed Approach for Healthcare Quality Improvement—Learning Through Developmental Evaluation

**DOI:** 10.3390/healthcare13030311

**Published:** 2025-02-03

**Authors:** Katharina Kovacs Burns, Marian George

**Affiliations:** 1 Clinical Quality Metrics, Data & Analytics, Alberta Health Services, Edmonton, AB T5J 3E4, Canada; 2 School of Public Health, University of Alberta, Edmonton, AB T6G 1C9, Canada; 3 Provincial Patient and Family Group, Alberta Health Services, Edmonton, AB T5J 3E4, Canada; mj.george@shaw.ca

**Keywords:** co-design, developmental evaluation, healthcare quality improvement, measuring experiences, outcomes

## Abstract

Background: Healthcare setting teams were challenged to understand how and what to measure regarding healthcare quality improvement (HQI), who should be involved, and what approach to apply. We aimed to determine if a generic co-design approach involving patients/families, multi-disciplinary care providers, and other staff was feasible to apply for HQI across diverse care settings. Developmental evaluation embedded in the co-design approach would determine its effectiveness, challenges, and other experiences across care settings and teams. Methods: Twenty-two acute and community care settings agreed to participate in applying a phased co-design approach to their HQI initiatives, including developmental evaluation. Each care setting team received co-design orientation and support. Semi-structured interviews and focus groups were conducted with patient/family advisors (PFAs) and care setting staff/care providers to gather their experiences with the co-design approach applied to their phased HQI work. Transcripts were thematically analyzed and triangulated with observation notes of care setting team discussions. Experiences were gathered from 17 PFAs and 68 staff/care providers across the 22 participating healthcare settings. Results: Themes for the orientation and each phase emphasized the importance of participants’ understanding, engagement, and ongoing open communication throughout the HQI co-design process. The orientation was viewed as key to facilitating good outcomes. Participants valued working together, gathering real-time experiences to “make a difference”, and having PFA voices involved in co-designing the HQI initiatives. Challenges were identified, including time commitment. Conclusions: Based on the overall developmental evaluation findings, there was consensus that a generic co-design of HQI initiatives was effective, feasible, and sustainable across care settings.

## 1. Introduction

### 1.1. Healthcare Quality Improvement—Challenges for Care Setting Teams

One of the fundamental goals of healthcare systems, leaders, and interprofessional care teams is to provide high-quality care and services to all patients when they need them. To meet this goal and be a well-functioning system, policies (global, national, and local), along with standards and procedures, have guided what and how practices and services are delivered [1,2,3]. Standards, also used for accreditation or measuring the performance of healthcare organizations, were viewed as drivers for healthcare leaders and staff to determine what was working and how well, as well as where improvements were needed.

Healthcare leaders and care setting teams have been challenged in their understanding of what measurements were needed to determine how successful they were at applying the standards for quality care. To start with, no universally accepted definition or description has existed for ‘quality’ in healthcare [1], although there are many studies that have explored different aspects or perceptions of what it should be. Some aligned quality with health services as being effective, safe, and people-centered [1,2,3,4], as measured through patient satisfaction, experiences, and outcomes. Others looked at quality in healthcare related to the health system, exploring essential attributes of being highly reliable, accessible, safe, timely, efficient, integrated, equitable, appropriately resourced, and focused on population health [1,4,5,6,7]. In addition to understanding what they needed to improve, another challenge faced by care setting teams was knowing how to go about it. Various published models and approaches [4,8,9] indicated their relevance for improving patient health care and service attributes, including safety and effectiveness, as well as healthcare provider competencies, care practices, and system-related factors [9,10,11,12,13]. Backhouse and Fatai suggested that we do not think about healthcare quality improvement as a single method or set of tools to measure the quality attributes, but rather as “a systematic continuous approach that aims to solve problems in healthcare, improve service provision, and ultimately provide better outcomes for patients” [10] (p. 82). The improvement shown through real-time measurement with patients/families and even care providers indicated that their experiences with certain care or services changed for the better or they benefited as a result of an intervention [4]. The quality care attributes were embedded in the experience measurement.

Although some suggested there was a difference between quality improvement for care or health services involving patients, their families, and their care providers, as well as the health system-level factors [14,15], others argued that there has always been an overlap between the levels. This is particularly true as care services and delivery are influenced or impacted by policy or practice decisions for continuity, coordination, and integration at the micro (clinical integration), meso (professional, functional, and organizational integration), and macro (system integration) levels [16]. This suggests that an integrated whole-system approach to healthcare quality improvement (HQI) and its sustainability is needed [16,17]. Therefore, a diverse team of senior healthcare leaders, care providers, other staff, as well as patients and families should be involved in defining the purpose of the improvement, along with approaches or strategies [1,9,18,19]. However, significant challenges with assembling and implementing diverse quality improvement teams were noted [20]. Identified issues included a lack of interest by staff to be involved in conducting or implementing HQI, a lack of understanding of what HQI is [21], an inherent resistance to change [22,23,24], and a lack of knowledge of where or how to start assembling diverse teams [18]. These issues have been associated with a variety of inadequate or absent system supports for quality improvement learning and implementation. Supports that were often lacking included leadership encouragement [1,21,22,25], a learning system culture to support workforce HQI skills training and capacity building [25,26], HQI or change implementation infrastructures and resources [23], and inclusionary approaches for system-wide and cross-discipline engagement as well as patient/family buy-in [1,25].

Addressing the issue concerning assembling diverse quality care teams therefore requires the right approach. It is critical to have the most appropriate people as part of the team involved at the outset to effectively plan, conduct, and evaluate their HQI initiative [25]. Orientation or training to build appropriate skills and capacity around HQI could also help [1,18]. One approach that embraces these concepts is co-design.

### 1.2. Co-Design—Benefits for HQI

Interest in co-design (also identified as co-production, co-creation, co-development, and collaborative participation) [26,27] expanded into what Palmer, Weavell, Callander, Piper, Richard et al. called the “Participatory Zeitgeist” or “the spirit of our times”, with emphasis on bringing the “users and choosers” of services and products together “to become makers and shapers” [28] (p. 21). Co-design models or approaches have evolved over the past two decades in different sectors and populations, including in healthcare. Some studies provided what they viewed as essential components of co-design or the outcomes related to actual healthcare improvements, while others focused on the ‘how-to’ approaches or toolkits [28,29,30,31,32,33]. However, there was no one standard or guide for a clearly and distinctly labeled, defined, or described co-design, but one thing was clear—there was the tendency to support active engagement or collaboration with patients/families and the integration of their lived experiences into the design or improvement of their health system and service delivery [34,35]. Interestingly, very few studies evaluated the effectiveness, value, or other attributes of the co-design approach, and none evaluated co-design as applied to healthcare improvement. Wang and Jiawen [36] conducted a literature review exploring co-design evaluation. They categorized evaluation in three ways, namely, by the approach or methods used, process or participant events, and outcome or results. Bate and Robert [37] suggested components of good design that could be measured, including “performance” or “functionality”, “engineering” or “safety”, and “the aesthetics of experience” or “usability” (p. 308).

A number of studies focused on experience-based co-design (EBCD) [38], with some targeting conditions or specific populations [39]. One study explored the emergent issues in EBCD participation through six different case studies, which showed how difficult it was to identify common or essential aspects of the process for improvement initiatives [40]. An evaluation of an EBCD program using a qualitative approach identified key challenges specifically related to patient/family volunteer recruitment and expectations related to their availability, engagement, and time commitments [41]. Strategies were also suggested for effective EBCD, including building on training and supports or having methodologies that prepared and developed the co-design capability of all participants. Two such applied strategies included the Mental Health Experience Co-design methodology, in which mental health service users and carers were involved in service planning, redesign, and evaluation [28], and the seven-step methodological framework called “A Generative Co-Design Framework for Healthcare Innovation”, used in determining how to improve care for children with chronic healthcare conditions and their families [42].

A capability framework for successful partnerships in QI suggested key knowledge, skills, and attitudes [43] for co-design. Workshops were viewed as essential strategies to assist patients and health professionals with learning new co-design skills, knowledge, and dispositions and for the successful co-creation of healthcare services [30,31] In addition, it was felt that care settings required support, such as staff with expertise to facilitate this work [44], and ‘practical resources’ [45] to help them implement and conduct their co-design work, particularly with the engagement of staff and volunteers. Thus, a clear understanding of facilitators and barriers is needed [46].

### 1.3. Study Aim

In this paper, we report on a study in which we piloted a co-design approach that was originally co-designed with other care teams to conduct their HQI work [47]. As part of this pilot, we utilized qualitative developmental evaluation to guide the evolution of this co-design approach with diverse teams within various acute and community healthcare settings or programs.

Based on our initial work involving co-design [47], we described it as being more than co-production or co-development. It was more about the people involved (having the right diversity, including PFAs), how and why they came together (establishing a care setting team to be involved in HQI), when they were involved (all members of team involved at the outset and throughout), how or to what extent they were involved (co-designing, co-planning, co-production of tools and HQI intervention, collaborating on data gathering and analysis, and co-reporting findings), and what they did or produced (made a difference in the co-designing of HQI for care settings and for patient and care provider experiences). We asked the following question: Is it possible to co-design a generic co-design approach that could be used by care setting teams in diverse acute, community, and other healthcare settings? Specifically, our goal was to explore the perceptions, experiences, and outcomes of individual patient and family advisors (PFAs), diverse care providers, and other staff (i.e., managers, administrators, quality improvement leads, etc.), as well as the care setting team as they collaboratively worked on their co-design approach for HQI.

In the co-design approach that we piloted in this study, we embedded developmental evaluation throughout as an iterative process. We started with the co-design orientation, followed by the co-planning, co-development, co-implementation, and co-evaluation of three phases of actions leading up to and encompassing HQI. In our final phase, developmental evaluation helped determine what care setting teams and, specifically, PFAs and staff/care providers generally valued or felt worked well and should be continued; which aspects they felt needed adjusting or more attention and why; if there were any notable differences or adaptations needed with the co-design approach by PFAs, staff/care providers, or teams in acute settings compared to community settings; and if our co-designed approach was perceived to be feasible and sustainable for HQI and the teams’ capacity-building actions.

## 2. Materials and Methods

### 2.1. Healthcare Settings and Participants for Co-Design in HQI Initiatives

The setting was Alberta Health Services, the largest integrated health system in Canada. Exploring HQI initiatives through different approaches across acute and community care settings was part of ongoing work within Alberta Health Services as applied through the Quadruple Aim, which included understanding the experiences of patients/families and care providers. For our study, some care settings were already part of a larger initiative in which PFAs were introduced to ‘real-time’ patient experience measurement as a way for them to reach out to patients/families to determine what mattered to them regarding their care [47]. Co-design was explored with these initial care settings and showed promise for being applied in other care settings. Hence, there was a desire to further explore the piloting of the co-design approach with a number of other diverse care settings and to test a generic approach. Other care settings were, therefore, recruited through an organization-wide newsletter outlining the co-design approach for HQI and conditions for participating. All care settings had to self-identify their interest in participating in and evaluating the piloted co-design approach intended for HQI initiatives. This was not a random or purposive recruitment approach as we wanted to offer an equal opportunity for any care setting to come forward. There was one condition. They were asked to commit to having diverse care providers and other staff (managers or leaders, administration, quality improvement consultants, and others as identified), as well as one or more PFAs involved as participants on their care setting team throughout the work.

In total, 22 care settings/programs were self-selected to be involved with the co-designed HQI initiative. Care settings included twelve acute or in-patient (two cardiac units, nine overcapacity cardiology units, and one surgery unit), seven community-based (five home care, one Adult Mental Health program, and one mid-transition program), and three ambulatory/out-patient settings. Each care setting had its own diverse team of participating PFAs, multi-disciplinary care providers, and various staff. A total of 17 PFAs and 68 staff/care providers were involved across all areas. Realizing that every care setting was unique and would always have varying degrees of challenges or issues to deal with (i.e., regarding their environment, staffing capacity and other resources, care delivery policies and procedures, and patient population and volumes), our initial discussions with each care setting team allowed us to transparently identify those challenges or issues. These needed to be considered and managed at the outset or throughout their co-design work. Although we could not anticipate what challenges each setting might encounter, we wanted to discuss some of the ones they felt could impact their team’s ability to follow through with co-design and HQI initiatives. Each team could determine its own approach to mitigate or manage any demand-capacity considerations to balance workload demands and impacts on co-designing HQI initiatives. For example, participating long-term care sites received COVID-19 pandemic policy changes as a result of high volumes of infected clients and staff. This put a stop to their involvement in the co-designed HQI work. As a result, these sites were not included in this review.

### 2.2. Co-Designed Approach for Healthcare HQI Initiatives

**Figure 1:**
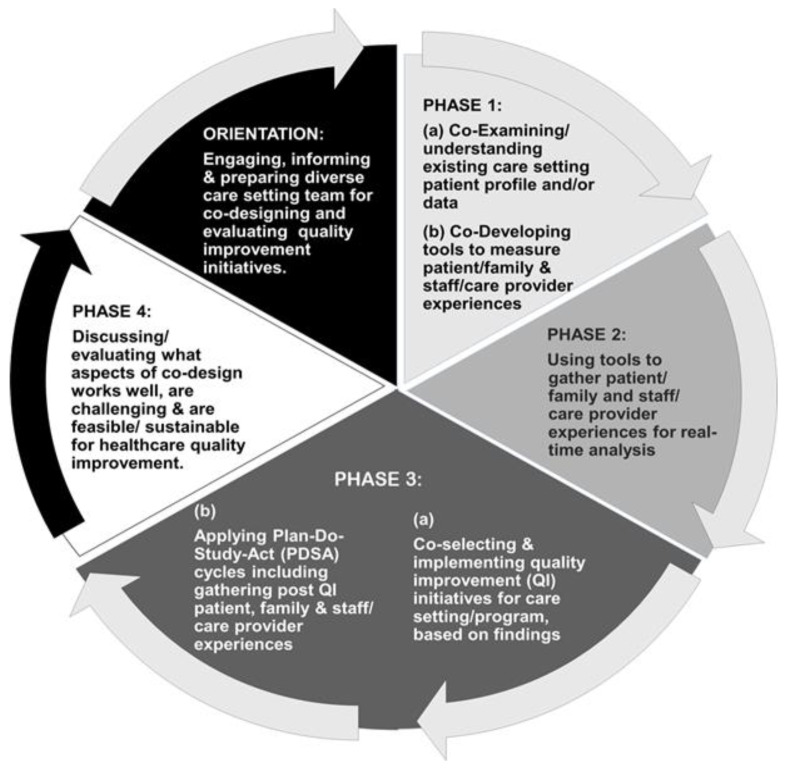
Co-designed approach for healthcare quality improvement.

Figure 1 illustrates the co-designed approach for healthcare QI we piloted and adapted through the developmental evaluation for general application across the various healthcare settings. Formally, we started with an orientation session. This was followed by three phases of specific actions, ending with the QI initiative involving Plan–Do–Study–Act (PDSA) cycles. A fourth phase was conducted to determine the overall co-design successes and challenges, as well as the feasibility and sustainability of continuing co-designing their healthcare HQI. This co-designed approach arose from the discussions and initial work the authors conducted in previous studies with care setting teams specifically around ‘real-time’ patient experience measurements [47].

During the ‘orientation’, team participants at each of the self-selected care settings were invited to a three-hour in-person workshop before COVID-19, which switched to two shorter virtual workshops during COVID-19. The intent of the workshop was to facilitate participant understanding of the co-design process and its relevance to measuring experiences and HQI initiatives; determine who needed to be involved in the team; and establish the team’s capacity to take ‘ownership’ of their co-design process as applied throughout the phased HQI work. It was during this orientation that each care setting team had the opportunity to define the setting’s uniqueness and consider tailoring aspects of the co-design approach for their HQI work.

To accompany the workshop, an orientation workbook, based on a standard template developed by the authors but tailored to care settings and participants, was provided to each participant. It contained key information about the team, including the contact information for their participating team members and details about the existing care settings, patient profiles, baseline experience data, the co-design process involving the three-phase approach for their HQI and anticipated outcomes, and exploring the co-design feasibility for HQI. The developmental evaluation of the co-design approach was built into the orientation and each phase, with consideration for PFA and staff/care provider experience mapping.

The co-designed Phases 1 and 2 concerned co-planning, developing, and implementing the patient/family and staff/care provider experience and outcome measurement tools prior to the HQI phase. Phase 3 included the co-selection and implementation of the HQI intervention/s based on the pre-QI experience and outcome findings. Implementation of the HQI initiative could involve one or more PDSA cycles along with experience measurements of patients/families and staff/care providers to determine what, if any, difference the HQI intervention made. In the final phase, i.e., Phase 4, follow-up discussions with PFAs, staff/care providers, and care setting teams were conducted regarding their co-design experiences with the HQI initiative. Benefits, challenges, limitations, staff and PFA resource needs, training or additional supports, and other aspects of the co-designed approach were also explored. This information was used to inform the discussions and perceptions regarding the feasibility and sustainability of applying co-design in HQI initiatives along with determining what, if any, continued or additional actions, supports, and resources would be needed.

### 2.3. Developmental Evaluation of Co-Design Approach

A phased participatory developmental evaluation approach [48] was utilized to guide the iterative development, testing, and validation of the co-design orientation (including workshop and guide) and four phases for HQI initiatives within the various care settings involved. Developmental evaluation was defined as “an emergent, exploring, learning-oriented and adaptive process in which those involved discover answers to their own situationally specific questions”; “is grounded in systems thinking”; and “supports innovation by collecting and analyzing real-time data in ways that lead to informed and ongoing decision making as part of the design, development, and implementation process” [49] (pp. 13–20). Through developmental evaluation, the intent was to examine and learn from the ‘real-time’ complex dynamic interactions and experiences of PFAs and staff/care providers with our co-design approach as applied in HQI [50]. We aimed to answer questions such as the following: What perspectives, experiences, and outcomes of PFAs and staff/care providers emerged during the co-designing of the HQI initiative that included the orientation and phases of the HQI initiative? How had the different values, perspectives, and experiences of PFAs and staff/care providers influenced or been influenced by the co-designing of the HQI initiative What did participants, generally, and, more specifically, PFAs and staff/care providers perceive to work well? What were the challenges or issues they identified as needing ‘real-time’ attention? Was anything around the co-designed approach for HQI different for participants within acute care settings compared to community care settings? Did participants perceive co-designing HQI initiatives as feasible and sustainable? Findings from these questions will inform the main study question—to determine if it was possible and feasible to have a generic co-design approach that could be applied across diverse care settings.

Based on the intent and questions to be explored as part of this study, a qualitative approach was incorporated into the developmental evaluation. The evaluation and related findings followed an iterative or real-time follow-up approach as applied to each phase of the co-design practice framework [30,31]. This included measuring experiences and impacts with the PFAs and staff/care providers involved as well as gathering their suggestions to improve any aspect of the co-design process that would help them follow through on the tasks leading up to (pre-QI), during, and following the implementation of the HQI initiative (post-QI). Supplementary Table S1 contains the predetermined questions covering various aspects of the co-designed orientation and each of the four phases. These questions were used as semi-structured guides for interviews and/or focus groups set up with care setting PFAs and staff/care providers and for discussions with the entire care setting team. These took place at the end of the orientation and each phase. Participant consent was obtained for audiotaping sessions. Otherwise, extensive notes were taken to capture verbatim comments. All recorded interviews, focus groups, and discussions were transcribed for rapid analysis.

### 2.4. Data Analysis

With the qualitative approach applied to the developmental evaluation, the intent was to have all PFA, staff/care provider, and team data analyzed rapidly and iteratively so that findings could be shared with the teams and used to inform any adjustments to the co-design process following the orientation and before each consecutive phase. All transcripts and/or notes were analyzed using descriptive thematic analysis [51]. One author used NVivo, and the other used a manual approach to code the transcripts and notes, with inter-rater checks conducted on the coding and emerging themes. Disagreements on codes or themes were discussed to reach an agreement on how to proceed. Data saturation was also determined when no new codes or themes emerged in the data coding for both groups [52]. These findings were also triangulated with additional detailed notes captured during care setting team discussions. As evaluations were conducted for each care setting, we shared their findings with them privately. For the purposes of this paper, we collated and merged codes/themes related to PFAs and staff/care providers across both groups and care settings, noting any unique findings between groups or between acute and community settings. Our aim was to report on general developmental evaluation data and results regarding the co-design approach for HQI initiatives.

## 3. Results

**Figure 2:**
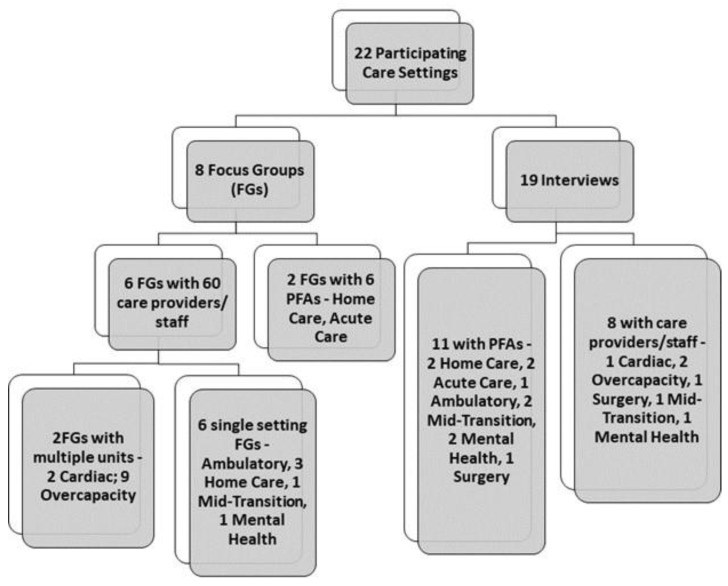
Participating care settings, PFAs, and care providers/staff.

Twenty-two care settings/programs participated in this study, with 17 PFAs and 68 care providers and staff participating in focus groups and interviews. Figure 2 provides the number of settings and participant focus groups and interviews.

The resulting codes and themes from all data sources reflected the experiences and perceptions of the majority of participating PFA and staff/care provider participants across the various care settings. Comments and quotes for each theme, however, were presented for each group (i.e., PFA and staff/care provider groups across all care settings) for comparisons of similar or contrasting findings. Any differences noted for acute compared to community care settings were described separately.

### 3.1. Developmental Evaluation of Co-Design Orientation

Regarding the co-design orientation that involved the workshop and workbook, the key themes that emerged across groups were ‘*being engaged*’, ‘*learning/understanding co-design*’, as in working together, ‘*having clear direction for the work planned/proposed*’, ‘*understanding time commitment, roles, and feeling comfortable contributing*’, as in conversations and idea-sharing, ‘*open communication*’, and ‘*co-design workbook as a guide*’. Supplementary Table S2 provides some relevant quotes for each theme as stated by PFAs or staff/care providers.

Although most participants originally expressed concerns that a three-hour workshop would be overwhelming, their opinions changed as the orientation session evolved. Generally, they felt they were *engaged* in relevant and meaningful conversations, working and learning together—“*the time for the orientation session flew by—all very interesting and intriguing as we were all interactively engaged in discussing the co-design process and understanding how this was going to unfold in our sites. It all seemed reasonable and exciting*” (care provider, acute care site). Some advisors in some settings observed that this was the first time that they and many staff were brought together, engaging in discussions about the issues, challenges, and needs concerning both patients and staff in those settings (“…*feels amazing to be at the same table talking about improving patient experiences in this home care setting*”—PFA community setting). For all those concerned, this was their introduction to co-design and its uniqueness, meaning more than just being engaged and more likely that they would be *working collaboratively* through all phases of work, with an end result of “making some small but important difference in patient care” (PFA, acute care). They all had to have the same understanding of what the plan was, *what was involved*, and what types of outcomes they were working towards. They understood that they had to *commit* to the work upfront, working as a team member and generally reaching agreement on every aspect of the work involved. This also meant that they needed to know how much time *commitment* was involved, something that many felt they did not really have, but they said they would make every effort to make the time—“*this process takes time and patience, more than I expected it would—I wasn’t sure what to expect really*” (PFA, acute care). Some felt unsure of the co-design process at the end of the orientation but trusted that they would have a better feel for it as work progressed. Hence, they were most interested in learning about their *roles* in the work leading up to and involving QI, including what tools they would develop and how, as well as the process they would use to gather ‘real-time’ patient and staff experiences related to the pre- and post-QI activities the teams chose to work on.

Since *open communication* was encouraged by the facilitators, participant interactions during the orientation workshop indicated that generally, both PFAs and staff/care providers *felt comfortable contributing* to the discussions. PFAs indicated that as patient advisors within a group that includes healthcare professionals, they often “*can feel intimidated*” (PFA, acute care setting). PFAs and staff/care providers in the community settings have never had this kind of opportunity; so, they found it exciting but also a bit overwhelming—“*as staff working in the community, we have not ever involved patients/families in our discussions about how to improve things…never really gave it much thought*” (staff/care provider, community setting). What helped make most participants feel more comfortable was the before-orientation informal introductions, icebreakers, and conversations. When held in person, food and beverages also helped. The organized professional facilitation of the sessions made the orientation process also feel safe and comfortable for people, allowing them to talk and share. PFAs cautioned facilitators, staff, and care providers against using acronyms or large medical terms—lay language was important if they were going to co-design the QI work. Some participants would have liked more *follow-up communication* immediately following the orientation, particularly around the timelines for getting started and the logistics for bringing their teams together with the facilitators to “*project-manage*” the co-planned/designed work from start to finish. Through the interviews, we also learned from participants that they appreciated the *co-design orientation workbook* as it provided them with information they could take away as well as reflect on throughout the duration of the pilot.

Feedback received from participants during the developmental evaluation of the orientation workshop and workbook was generally positive and favorable. PFAs were glad to be “*bringing their own voice and expertise*” to the co-design process, and most participants felt comfortable with the proposed approach and plans—“*Having the big picture helps to understand how the process will unfold*” (Staff, acute care). Minor changes or adjustments were made to the orientation and workbook based on what we heard from participants. With each care team orientation session, comments about the process and content became more positive.

### 3.2. Developmental Evaluation of Co-Design During Phase 1

Phase 1 consisted of two parts. During Phase 1 (a), the participants were introduced to and engaged in a focused review and interpretation of either existing data for their care setting or from the literature regarding similar care settings, followed by (b) the co-development of measures/tools to gather both patient and healthcare provider experiences based on care setting team discussions or areas of issues identified from the setting data (i.e., poor patient experience ratings). Themes for this phase, based on PFA and staff/care provider comments regarding the co-design efforts, included having a ‘*clear understanding of the care setting and issues*’, ‘*interpreting data*’, *or* ‘*making sense of the data*’ to identify what worked well and what areas needed further exploration and possible interventions, as well as ‘*framing relevant experience questions*’, ‘*creating reasonable experience measurement surveys*’, *and* ‘*exploring experiences with COVID*’. Supplementary Table S3 provides some quotes for these various themes.

Although most team members said they originally believed they had some ‘*understanding of the care setting context and issues*’, those in care settings that had existing patient experience data gathered as part of the standardized tool known as the *Canadian Patient Experience Survey—Inpatient* Care (CPES-IC), and other outcomes were surprised to see these data available on dashboards. Most teams admitted that they did not know this system-wide measurement data existed for the majority of acute care and even some community care sites across [anonymized]—“*We did not know that any data existed for our unit!*” (staff/care provider, acute care). The data reviewed with each of the teams guided their discussions to ‘*interpret the data’ or ‘make sense of the data*’, identifying key patient care/experience issues (i.e., where patient experiences were rated as very low to the lowest using ‘always’ or ‘satisfaction’ ratings). These issues were discussed with teams as potential areas they could focus on to explore further through ‘real-time’ surveys as well as for their planned HQI initiatives.

During Phase 1 (b), participants created a fishbone that identified clusters of issues that were later themed. These themes, or domains, guided participants with ‘*framing relevant experience questions’* for issues they wanted to explore in more detail. For settings involved in this process during COVID-19, participants wanted to also ‘*explore experiences with COVID*’ and the related impacts or consequences for patient and staff experiences regarding care/care delivery, care transitions, and other aspects. Teams discussed and sometimes debated which questions were most relevant to ask patients/families and staff/care providers so that they could compare responses between the groups. They found it time-consuming but valuable to be ‘*co-creating reasonable experience measurement surveys*’. The PFAs made certain the “*patient experience voice was heard*” or that “*what matters to patients*” was applied throughout their discussion. They did not want the questions to reflect the perspectives of staff/care providers or be in their “clinical language”.

Many participants could see that it took time and patience to develop reasonable experience measurement surveys—“was surprised at how long it took us as a group to select our questions and create a reasonable survey which we could use as we talked with patients on the unit” (PFA, acute care). COVID presented additional challenges for teams and for doing the co-design work virtually—“Working together to create a survey during COVID was challenging—never sure who would be able to take part in the discussions, as our team, especially staff were asked to work on COVID units” (staff, acute care). Teams really needed to be encouraged to keep going even when only a few people could attend the virtual meetings. The surveys were shared with others for feedback—“everyone had an opportunity to share thoughts on the questions and survey when done” (PFA, community setting).

Some community (mid-transition, community mental health, and ambulatory) settings did not have previous patient and care setting profile data, and co-designing things was more challenging for the team—“*It is more challenging to understand a community program which has no data regarding the care or support provided, or experiences of patients/clients and their families. We are talking with staff in this project needing to now be open and honest about what needs to be measured and why*” (PFA, mental health community program).

Based on the developmental evaluation of this phase, most teams felt the co-design process involved in this phase worked well, considering that many individuals had no experience with interpreting existing data or designing surveys to gather experience data—“*impressed with the process of developing the surveys—worked well*” (care provider, community setting). The time commitment of participants for this phase needed to be more appropriately estimated.

### 3.3. Developmental Evaluation of Co-Design During Phase 2

When the surveys were developed as part of Phase 1, everyone was eager to move forward with getting things in place to start gathering data and seeing some results. The themes included ‘*felt supported in care settings to gather data*’, ‘*gathering real-time data—what matters to patients*’, and ‘*analyzing and interpreting experience data in real time*’. Supplementary Table S4 provides some quotes/comments for each of the themes.

Participants said they ‘*felt supported by care settings to gather data*’—setting staff, care providers, and managers were receptive to having PFAs come in and talk to patients/families about their experiences with their care—“…*this process of having patient/family advisors working with us to gather and understand what our home care patients think and experience about the care we deliver is a great idea, but some staff are feeling a bit uncertain of this process as well*” (staff/care provider, community care). Most staff/care providers appreciated the fact that the PFAs were ‘*gathering real-time data—what matters to patients*’ and that they were part of the study team ‘*analyzing and interpreting the experience data in real time*’. PFAs clearly indicated that this experience with gathering real-time experiences from patients/families as a member of the care setting team was empowering—“*I felt I was not only a peer on the study team but also a peer patient/family member who could be trusted to ensure I would bring the patient voice to the care setting staff/care providers and take part in making a difference*” (PFA, acute care). These data would provide staff/care providers with evidence that they were somewhat reluctant to have but that they were curious about and wanted to see. They anticipated that PFAs would be trusted by patients/families they spoke with. Patients/families would be honest in talking about what was good about their care and what could be improved. PFAs were doing this part of the work alone, and at times, they felt that the time they contributed was somewhat taken for granted by everyone else on the team as they waited to see the survey findings—“*Being a volunteer, I did not have time constraints and could listen to them. At times, I wasn’t sure that the home care pilot staff or facilitators were aware of the time that the surveys actually took to complete*” (PFA, home care).

Care setting staff/care providers admitted not being aware of all that PFAs did regarding gathering data, but they certainly expressed their appreciation when the results of the data were shared. They were also glad to see staff/care providers involved in co-designing the surveys and analyzing the results. They were especially pleased to see accolades as well as areas of concern in the patient experience data. Also, most care setting staff, care providers, and managers were supportive of the study team working with them to complete the online survey specifically developed to gather their perspectives and experiences. “*It’s not often we get to be part of a group developing a survey for us—asking us about our experiences with talking with patients and families and where we see things could improve with and for our patients*” (staff, community care). The differences in responses by PFAs and staff/care providers to similar questions made for good discussions in some care settings and prepared them for Phase 3.

### 3.4. Developmental Evaluation of Co-Design During Phase 3

Participants at each care setting were involved in interpreting areas of possible improvement identified within the data gathered in Phase 2 and applying that in Phase 3 (a), where they co-selected and implemented QI for their care setting, and in Phase 3 (b), where they gathered post-QI experience data to see what difference the QI intervention made, as part of the Plan–Do–Study–Act (PDSA) cycles. Common themes across Phase 3 activities included ‘*understanding QI*’, ‘*having a flexible approach*’, ‘*engaging in QI planning decisions*’, ‘*comparing data for pre- and post-QI*’, and ‘*making a difference*’. Supplementary Table S5 provides some quotes and comments from PFAs and staff/care providers.

A natural follow-up from Phase 2 was the discussion of what the data were telling participants about the care setting and what patients/families and even staff/care providers understood to be working well and needed improving. This aligned with discussions about *understanding QI*, the need for it, and their expectations for changes—a conversation they all started to have during the orientation session.

Co-designing a QI plan for the care setting required more intense discussions about the intent of the QI initiative, strategy, goals, and timelines, as well as a communication plan. A common theme that came out of interviews or discussions was their appreciation for *having a flexible approach* to how participants selected their QI intervention to address an identified issue or for the PDSA cycles involved in QI. How and when participants became *engaged in the QI planning decisions* was identified as crucial, particularly for PFAs, as they could finally see how they could contribute to the QI decision process based on what they heard patients/families in the care setting tell them about what improvements in their care were needed. Staff/care providers appreciated that there would be an agreed-on plan for what the QI was and how it was going to be implemented and monitored. There was joint ownership of the plan. “*Never thought we would be doing PDSA cycles for QI with patient/family advisors who could tell us firsthand what our clients said we needed to improve—this is a first for us and a huge learning opportunity!*” (care provider, community setting).

PFAs also *felt well-supported* by care setting staff and the pilot coordinators to gather post-QI patient experiences and analyze and interpret rapid real-time data as part of the PDSA cycles, which then allowed for *comparing pre- and post-HQI experience.* If differences between the pre- and post-QI datasets showed improvements in experiences following the QI implementation, they felt they were *making a difference* with HQI in the care setting. Staff hoped they could continue *making a difference* around other patient or practice issues that were still identified as needing improvement but not part of the current study HQI. “*I fear that while we had a great opportunity with the co-designed HQI activities, we will lose some momentum applying this whole approach as we try to follow up on the next areas needing improvement*” (staff/care provider, ambulatory care). Staff and PFAs generally felt good about the co-designed HQI process and plan and hoped that their co-decision regarding the HQI made or continued to make a difference in patient experiences and outcomes.

Some PFAs said they would have felt more comfortable about this phase if they had received more direct communication and encouragement from the pilot coordinators and care setting staff/care providers throughout as this phase took a lot more time than anticipated, much of it “waiting time” as adequate time was needed for the HQI intervention to be implemented and monitored. They wanted to be sure that things did not stagnate while the HQI was being implemented and that what they were doing as part of the HQI and post-HQI was making a difference for patients as well as care setting staff/care providers.

### 3.5. Developmental Evaluation of Co-Design During Phase 4

When all was said and done with the HQI work, participants shared their experiences and suggestions around the feasibility and sustainability of applying co-design as an integral part of their phased healthcare QI initiatives (Phase 4). Common themes included ‘*communication throughout*’, ‘*valued experiences*’, and ‘*benefits*’ associated with the overall co-design process in QI initiatives, as well as some ‘*limitations*’/‘*challenges*’, with a consensus that ‘*co-design is needed and feasible*’. Supplementary Table S6 provides some comments and quotes for these themes.

Everyone indicated that the key to the successful implementation of the co-design approach through the orientation and three phases was the timely, informative, and respectful *communication throughout*—“*communication was the key to making or breaking acceptance of the co-design approach*” (staff/care provider, community care). This included communication from the facilitators or “pilot coordinators” to PFAs and staff/care providers during meetings and specifically with activities involved in the orientation and each phase. Everyone felt included in the discussions, listened to, encouraged to contribute, and respected for their opinions and suggestions. The communication kept everyone informed and on track with what was happening throughout. Communication was also felt to be significant between PFAs and the staff/care providers—there were key moments when participants felt that open communication, including disagreements, made them appreciate each other’s reflections on things. PFAs particularly felt they were actually at the same level of engagement in discussions or making decisions as their staff/care provider team members or “partners”. The latter was also reflected by participants in what they said about their “*valued experiences*” with co-design, personally and as a group. They indicated their appreciation for the opportunity to be involved in the co-design process and resulting work. PFAs claimed that one of their most valued experiences was being “one of the team”, actively contributing to developing and implementing all aspects. Staff/care providers noted the added value of having PFAs involved in the QI work, which they felt added credibility to efforts to improve patient experiences and outcomes.

*Benefits* were noted around working together towards the same aims in HQI, particularly gathering and listening to the patients’ voices in real time to drive QI. Co-design was a new way of learning and doing this together, likened to what Patient- and Family-Centered Care really means. However, participants also identified some *challenges or limitations* with co-design, including that it took more time to do it right, that people had to be committed to make the time to see it through, that everyone in the care setting understood and had buy-in for the co-designed QI process, and that there were available “ready advisors” interested in being involved in this type of work.

Generally, all participants supported the co-design process and felt that it was *needed and feasible*, as well as sustainable. Not only did PFAs believe co-design for HQI was good to have and continue at AHS but their experience from the pilot initiative also demonstrated that the “*cost is low but the value is huge*”. Staff/care providers also felt that the process should continue across all areas within AHS and that more training to build team capacity for co-designed HQI within care settings would make a difference in healthcare safety and overall quality.

Even with challenges noted during COVID, when restrictions to care settings were in place across acute and community care settings, PFAs and staff continued to support the process. They regrouped, and where possible, PFAs were supported by care setting staff to contact patients/families virtually, thus continuing the effort around the HQI and, including COVID as part of the investigation, to learn from patients and families directly regarding what they needed or what worked well.

Participants stated what they felt were key actions for making co-designed HQI successful and therefore more likely to be feasible and sustainable—‘early relationship building—trust’; ‘orientation’ to the process; ‘clear roles/responsibilities’; ‘collaboration’; ‘communication throughout’; ‘more advisors needed for backup’; ‘additional preparation/training (for advisors) and additional staff/care provider preparation for advisors’ arrival’; ‘ownership of a different process’; and ‘facilitation—guidance and support’. As co-design was a new process for all of the care settings and teams involved, the developmental evaluation provided an essential guide to allow these teams to determine what key actions were working well and what more was needed to support the team to be successful. Teams indicated that this work needed to continue and be spread and scaled to other settings.

## 4. Discussion

As part of their HQI work and study over two years, the authors introduced a new co-design approach that was, in fact, co-designed with several diverse acute and community-based care setting teams [47]. This initial work was instrumental in informing this study to further pilot the co-design approach with more diverse acute and community care settings as part of their HQI work. In addition, real-time qualitative developmental evaluation was applied to determine what the care setting teams felt and experienced as a result of the co-design approach applied to their HQI work. Our aim was to determine if, through broader and more diverse piloting and evaluation, we could frame one generic co-design approach that would be applicable in any healthcare setting. Although there are a few studies in the literature that have evaluated their co-design approaches [36,37,38,39,40,41,42], none utilized developmental evaluation throughout the process, and none demonstrated the co-design application in specific healthcare initiatives such as HQI. As a result, our study presents some unique methodology and findings.

Although our approach to co-design applied in HQI work was unique, the literature provides some similar actions or elements, particularly with the how-to approaches [29,30,31,32,33]. Inherent in the co-design approach was the opportunity for care settings to assemble appropriate diverse HQI teams of PFAs, care providers, QI and other staff, and managers/leaders [1,9,18,19,23]. The co-design orientation provided each team with the opportunity to storm and norm their understanding of HQI and what it meant or would mean to them, including their willingness to break old habits such as resisting change [22,23,24]. They engaged in learning about the benefits of HQI with the purpose of improving the safety and quality of care for patients in the care setting. This meant they would learn skills and/or develop the capacity to measure the experiences of patients through the lens of “what matters to patients” [25,26]. While working towards improving patient experiences, each team also needed to understand the parallel experiences of care setting staff/care providers and system factors, including relevant policies or procedures. As a result of this unique 360-degree evaluation lens of the co-design approach, each member of the diverse teams began to understand how to work better together as a team and with each other. They also learned more about each other, including their concerns (i.e., stresses, burdens, and demands), needs, and roles in healthcare and care improvement, to be more respectful of demand-capacity issues facing care providers and staff, and to show more resilience when sharing workloads or adjusting HQI work and timelines. The literature does not describe this type of comparative analysis of experiences across different care setting team members. This study will contribute to this aspect of the literature.

The developmental evaluation provided the opportunity to explore and directly apply the experiences and perceptions of PFAs and staff/care providers to continuously improve on the co-design approach for HQI as implemented in various diverse acute and community-based care settings within a large healthcare system. These settings became the incubators for the piloting of this new approach while measuring the direct perspectives, experiences, and impacts of those involved. We were able to determine generally what participants valued or felt worked well with the co-designed approach and should be continued; what they felt could be emphasized more or improved; what they learned from the perception or experience differences between PFAs and staff/care providers as part of teams or between teams in acute compared to community settings; and what capacity-building actions and resources/supports needed to be in place for care setting teams to be successful with co-designing their HQI and ‘making a difference’.

Generally, our study confirmed but also expanded on what the few other studies found regarding the evaluation of the aspects of co-design in healthcare. This included the need for good communication and other participatory approaches for effective co-design [32], with workshops (or in our case, orientation) being essential for learning skills, knowledge, and dispositions [31,40], and understanding the enablers along with limitations or challenges with co-design (including the time involved and preparation of participants and care settings for co-designing all aspects of the work), as well as resources to support co-design [46]. In addition, our study provided two unique aspects—one was the co-design approach for HQI involving phases of activities. The other was the developmental evaluation, which informed in ‘real time’ what things worked well for care setting teams as they worked their way through the co-design orientation, three phases leading up to and encompassing the HQI, and a fourth phase examining the feasibility and sustainability of the co-designed approach for HQI. The approach and findings will contribute to the literature on co-design and its evaluation.

Throughout the evaluation of the orientation and four phases, both PFAs and staff/care providers emphasized how they began to see the value of co-design—i.e., working, learning, and understanding together as they planned and implemented all aspects of the HQI work. Essentially, we found that co-design facilitated a clear culture shift for many of the participants involved in their care setting work, with individual and group buy-in or ownership of the process, including co-designing their measurement tools for their real-time experience and outcome measurement for pre- and post-HQI initiatives. They felt the costs related to co-design were relatively low, including the time involved by staff/care providers, which normally would be accounted for as part of their practice improvement. However, participants also suggested that there be certain considerations or criteria in place. For example, they felt that the team should agree to and openly discuss and/or work through any immediately identified challenges or issues in real time, as part of the co-design process. Everyone owned the process and the outcomes—there would always be things to deal with regularly as part of any care setting. This attention to openly identify and manage issues as they crop up would help ensure the success of co-design implementation, particularly as part of their HQI work. Most participants in this study, similar to those in the literature, felt that these things could only occur when everyone on the team was actively engaged in all of the processes, contributing, sharing ideas, and making suggestions to each other [32].

Generally, the perceptions and experiences of PFAs and staff/care providers for both acute care and community settings were similar. One difference noted by PFAs working in community-based settings was that it took more time for them to connect with and survey patients/families in person or virtually. This time commitment was not always initially acknowledged by their teams or other setting staff/care providers but needed to be brought forward to team meetings.

Although further studies could be suggested regarding the application of our co-design approach in many other diverse care settings or even similar care settings across one or more health systems, we felt that our study answered our initial study question and aim, confirming that a generic co-design approach was plausible and feasible to develop and apply as part of HQI initiatives across diverse care settings. We especially felt that the application of developmental evaluation provided the results to confirm our question or aim. We cannot generalize about the different HQI initiatives and their results as that was not the aim of this study. These latter findings have been shared in other synopsis reports, publications, or presentations.

Based on the results, we also felt that there could be broader practical implications for the application of this generic co-design approach, including enhanced care setting team capacity building through the real-time co-design and evaluation process (evaluation should become part of the phased co-design process); care setting and team ownership of improved or better care and care delivery (diverse teams should drive HQI); increased confidence of staff (staff supported by others in what they do or what should be improved in care settings, resulting in improved patient experiences and outcomes); PFAs feeling they have a voice (PFAs were part of the care setting team and meaningful contributors to the improvement of care and care delivery); and application of the generic co-design approach not only in healthcare but also in other sectors (e.g., social services, rehabilitation, or businesses). Other implications or lessons learned for co-design application during the COVID pandemic included teams virtually conducting co-design for HQI or other initiatives; study teams conducting virtual developmental evaluation measurements with individuals or teams through interviews or focus groups; and co-design teams providing support or taking over HQI initiatives for staff or care providers so they could focus on more urgent patient issues or other challenges. Perhaps the co-design approach could be used by care setting or system leaders to frame more appropriate strategies to mitigate demand-capacity issues, especially during pandemics.

Other comparative studies could involve the application and evaluation of the co-design approach with diverse care settings across other health systems, jurisdictions, or countries or in similar care settings across one or more health systems, jurisdictions, or countries. Studies should also involve comparisons across rural versus urban care settings. An experimental study could also be proposed to compare randomly selected diverse care settings to control settings, accounting for different variables across them and applying the co-design approach to experimental settings while control settings are applied to other approaches for HQI. This type of study would further our understanding of different perspectives and experiences of care setting teams in applying the co-design approach versus others. Which would they prefer and why?

Working with diverse care setting teams evaluating a co-design approach as applied to HQI was not without some limitations. One limitation of our study was that all care settings had to self-select to participate in this initiative as there needed to be interest and commitment. Participant perspectives and experiences could therefore be perceived as being more positively biased towards the co-design approach from the outset. Another limitation was that we could not manage or anticipate all challenges and issues experienced daily or regularly in care settings. In our study, 6 of the 22 participating care settings had to alter approaches and timelines because the COVID pandemic created organizational stresses on the workforce or care settings. Specifically, in long-term care settings, the co-design work was put ‘on hold’, while in another setting, COVID impacted the choice of HQI intervention made by the team because administrative challenges had to take priority. We talked through those situations and considered what impact, if any, they would have on the experiences of the care setting team and individuals applying the co-design approach as part of their HQI work. These experiences were captured and included in our results synopsis. We note that although we cannot generalize across HQI initiatives and outcomes because of challenges that might impact their implementation, we were able to determine that a generic co-design approach was still possible and feasible to apply across diverse care settings. Other adjustments during COVID, such as working virtually, allowed teams to conduct their co-design and related applications within various care settings over longer timeframes. Some of the team members also managed to take over this work, leaving more urgent patient care (such as COVID) to be attended to by staff and care providers. This could be viewed as a balanced strategy, which addresses some of the demand-capacity issues that can occur on occasion, especially during pandemics. However, further studies, discussion, and analysis are needed to examine co-design and demand-capacity balance generally as well as specifically in relation to pandemics.

## 5. Conclusions

Through the developmental evaluation of our piloted co-design approach for HQI, we were able to show that the approach can be successfully implemented and evaluated with any real-time adjustments made to the processes with care teams as needed. Overall, the evaluation of PFAs and staff/care providers in the diverse care settings indicated that their experiences were positive. Although experiences for PFAs and staff/care providers revealed some differences, they generally appreciated the co-design approach as it gave each group the sense of being equally part of the care setting team and meaningfully involved or contributing to all phases of their HQI initiative. They also learned to trust and value each other as part of their team. Perhaps co-design helped them build resilience to openly talk about and implement real-time strategies to mitigate the impacts of demand-capacity issues affecting both patient care and the well-being of care providers and staff.

Based on the findings, a generic co-design approach was felt to be feasible for application in both acute and community-based settings. Also, based on the developmental evaluation of the co-design approach, the authors published a generic co-design approach [19]—this was intended to be a guide to support the spread and scale of co-design amongst other care settings and teams. The intent was to see this co-design approach grow, evolve, and spread across diverse settings and be supported through ongoing co-design resources, such as the orientation guide and real-time evaluation. Further studies have been suggested regarding the application and evaluation of the generic co-design approach. Broader implications for the application of this generic co-design approach should include its capacity-building opportunities within healthcare as well as in other sectors beyond healthcare.

## Data Availability

Because of the privacy and confidentiality considerations for the data gathered from advisors and others, raw data cannot be made publicly available or shared. Requests can be made to the corresponding author for some of the de-identified or analyzed data.

## Supplementary Materials

The following supporting information can be downloaded at: https://www.mdpi.com/article/10.3390/healthcare13030311/s1, Table S1—Semi-Structured Focus Group/Interview Question Guide for PFAs, Care Setting Staff/Care Providers, and Teams Regarding Co-design; Table S2—Themes and Participant Quotes during the Developmental Evaluation of the Orientation to Co-designed HQI; Table S3—Developmental Evaluation Themes and Quotes from Participants Regarding their Co-design Experience during Phase 1; Table S4—Developmental Evaluation Themes and Quotes from Participants Regarding their Co-design Experience during Phase 2; Table S5—Developmental Evaluation Themes and Quotes from Participants Regarding their Co-design Experience during Phase 3; Table S6—Developmental Evaluation Themes and Quotes from Participants Regarding their Co-design Experience during Phase 4.
