# Supplementary material for: Exploring a Co-Designed Approach for Healthcare Quality Improvement—Learning Through Developmental Evaluation
Source: Healthcare (Basel). 2025 Feb 3;13(3):311. doi: 10.3390/healthcare13030311 (PMC11817868; doi:10.3390/healthcare13030311)
Supplement: Supplementary file 1 [file healthcare-13-00311-s001.zip › Supplementary Table 1 - Semi-structured Focus Group and Interview Question Guide.pdf]

Supplementary Table S1: Semi-Structured Focus Group/Interview Question Guide for PFAs, Care Setting Staff/Care Providers and entire Setting/Site Teams Regarding Co-design Approach for Healthcare Quality Improvement (HQI)

Regarding Co-design ORIENTATION
**1. Regarding the orientation workshop - how effective was that orientation session along with the workbook, in preparing you for 'co-design' work? For the pilot HQI work? Your role? Generally?**<br>o *How did you understand your role to be with co-designing this HQI pilot?*<br>o *Were you comfortable with the co-design approach taken for the pilot work in the orientation session? In understanding HQI and care setting issues? In HQI planning? For the implementation of the co-designed HQI plan in the care setting? Overall?*<br>o *Was there any additional information or preparation you feel would have been beneficial to have had during the orientation workshop?*<br>o *Did you find the orientation guide and presentations helpful/beneficial/useful?*<br>o *Was there adequate opportunity for your input and interaction regarding the different pilot phases and processes?*<br>o *How would you describe your experience working with setting/site/program team (i.e. with a mix of PFAs, staff, care providers, managers, others)?*<br><br>**2. What, if anything, could be done differently or improved on to prepare you for co-design work in your area/unit/program?**<br>o *What would you have liked to see or have more of?*<br><br>**3. What is your key take away from participating in this co-design HQI orientation?**<br>o *What were some of your best learnings?*
**PHASE 1: Regarding Co-design of Care Experience Measurement Surveys**
**4. Overall, how effective was this co-design approach for identifying and understanding:**<br>o *Existing care setting data or similar literature data and what they tell you?*<br>o *Key issues for care settings, care providers and patients? i.e. driver or fishbone exercise identifying issues?*<br>o *developing experience measurement survey tools to gather relevant patient/family and staff/clinician experiences in real time?*<br><br>**5. What was/were learning moments for you partnering with other PFAs and staff/ managers in discussing care setting data and issues and co-designing relevant patient and care provider experience survey questions?**<br><br>**6. Specifically, regarding your role and/or involvement ….**<br>o *How did you feel about your capacity to actively be involved in discussions and developing the survey questions/tool?*<br>o *Any comments or suggestions related to this work, your role, and how we can improve on co-designing real-time experience measurement initiatives?*

PHASE 2: Regarding Using the Co-designed Experience Measurement Surveys to Gather Real-time Experiences

7. **Overall, how effective was this co-design approach for using experience measurement survey tools to gather patient/family and staff/clinician experiences in real time?**
   - *What was/were learning moments for you partnering with other PFs and staff/ managers in gathering and analyzing data in real time?*

8. **Specifically, Regarding Co-design of Care Experience Measurement (Pre HQI) ….**
   - *What are your thoughts on co-designing experience surveys and data collection?*
   - *Any comments or suggestions related to the care experience measurement work, your role, and how we can improve on co-designing real-time HQI experience measurement initiatives?*

PHASE 3: Regarding Co-selection, implementation and follow-up on pre and post HQI

9. **Specifically, Regarding Co-design with HQI Initiative and Post HQI Measurement….**
   - *What are your thoughts regarding how we co-designed the HQI issues-framing and selection of the HQI intervention based on the Pre HQI patient and staff/care provider experience findings?*
   - *What are your thoughts on the discussion of the co-designed interpretation of the pre-post HQI findings, and follow-up?*
   - *Any other comments or suggestions related to the HQI work, your role, and how we can improve on co-designing QI initiatives?*

PHASE 4: Regarding Feasibility & Sustainability of Co-designing HQI Initiatives

10. **Thinking back to the beginning and the orientation to co-design and specifically with HQI, do you view co-design differently now? If so, please describe.**
    - *What are your thoughts regarding the co-design approach we used throughout the pilot phases and activities – i.e. partnering with Patient/Client or Family Advisors/Volunteers and other staff/managers?*
    - *What were some outcomes from the overall pilot you think were worthwhile & could be shared?*

11. **How sustainable or feasible is this type of co-design process for healthcare HQI?**
    - *How feasible is co-design for staff and PFAs to implement at settings/sites for HQI initiatives?*
    - *How did co-design process impact the HQI initiative development and outcomes?*
    - *Is co-design for HQI initiatives sustainable in the long term?*
    - *What were some benefits of co-design in HQI initiatives?*
    - *What are some challenges we need to consider for future or ongoing initiatives?*
    - *What needs to be in place in order to sustain co-design projects?*

12. **How can we improve on supporting everyone better in a co-design process?**
    - *What suggestions do you have for improving co-design to be easier for health care teams in different settings/sites to implement?*

13. **What is your key take away from participating in this co-design QI pilot?**
    - *What were some of your best learnings?*

14. **What would you have liked to see or have more of?**
