# Supplementary material for: Exploring a Co-Designed Approach for Healthcare Quality Improvement—Learning Through Developmental Evaluation
Source: Healthcare (Basel). 2025 Feb 3;13(3):311. doi: 10.3390/healthcare13030311 (PMC11817868; doi:10.3390/healthcare13030311)
Supplement: Supplementary file 1 [file healthcare-13-00311-s001.zip › Supplementary Table 2 -Themes and Participant Quotes re Codesign Orientation.pdf]

Supplementary Table S2: Themes and Quotes from Participants Regarding their Experiences with the Orientation to Co-design

Themes	*Participants*	*Quotes*
**Being Engaged**	PFA	"…all engaged in exploring issues, existing data/evidence, challenges and approaches for change"; "Don't believe that the staff know what a patient advisor can offer or what they can do… education needed for staff to understand the reasons for patient advisors".
	Staff/Care Provider	"…working together on improvement solutions based on the data"; they were "examining outcomes together".
**Learning/ Understanding Co-design**	PFA	"co-design offers ways to contribute as an advisor";<br>A few PFAs felt the opposite with "too much information to absorb" and "did not get a feeling for co-design at orientation but [was] learned as [we] went along due to inclusiveness to all decisions and development of tools".<br>"I think you provided the background of patient and family engagement and how this was another way that patients and families could provide or assist in improving health care and that this project was sort of to prove the concept that using patient and families to assist QI was feasible and positive".
	Staff/Care Provider	"…co-designing for QI is of benefit – works well with working group of staff and advisors – a coordinated team effort… structure is then easier to provide and follow".
**Having clear direction for the work planned/ proposed**	PFA	"Need to ensure everyone understands what the goal or aim is";<br>"by end of orientation, we were well prepared to know what was coming and be prepared for co-designing the work"; "overwhelming at first, but everything was well laid out, organized"; "We knew what the purpose of the work was and the overall goal, and the steps we needed to complete to get there"; "once I saw the direction, could settle down".
	Staff/Care Provider	"pilot is clearly aligned with unit goals to have a process for QI that we can duplicate – is easily planned and implemented"; "Everything was so well organized, coordinated and presented – makes it easy to see what we will be doing as a team and hoping to accomplish as a result"

**Understanding Time Commitment**	PFA	Essentially understanding "the big picture";<br>"…lots of work to do [in this pilot] but all exciting!"<br>"need to clarify the time commitment for each phase and overall initiative".
	Staff/Care Provider	"…essential to understand how much time will be involved for staff and care providers on the unit. Either we try to dedicate staff to this initiative or rotate staff to be involved – both may need to happen and may be good learning opportunities as well".
**Roles**	PFA	"…pilot information was important for Advisors to understand what they would be doing and what they are getting into";<br>"Your call after the orientation allowed me to respond or ask questions about the pilot and my role".
	Staff/ Care Provider	"… will need to be more open and conscientious about Advisors working alongside us in QI activities… we are not doing this alone".
**Feeling Comfortable Contributing**	PFA	"Initially it felt overwhelming, but due to the professional and organized presentation I was able to become comfortable in the group";<br>The workshop "created a safe place … felt comfortable contributing"; and "functional/helpful";<br>"When you prompted us to respond at committee meetings related to our opinions, I think it helped us provide our voice to the discussions";<br>Advice "cautioning professionals about the use of medical terminology with layperson volunteering".
	Staff/Care Provider	"the facilitation made it easy and comfortable to contribute"
**Open Communication**	PFA	Facilitators to follow up frequently "just as encouragement"; "don't assume we understood or got everything – check in with us"; "really good – we were encouraged to give our client perspectives so that would not get lost"; "we established a good meeting ground".
	Staff/Care Provider	"stopping us and asking questions throughout [workshop] to keep us engaged is a good approach – keeps communication open"
**Co-design workbook as a guide**	PFA	"The orientation guide really, really worked well! ...used throughout the pilot".
	Staff/Care Provider	'…liked the total approach – very easy to follow";<br>"workbook keeps everything in one place for me to refer back to"; "notes are all in one place".
