# Supplementary material for: Exploring a Co-Designed Approach for Healthcare Quality Improvement—Learning Through Developmental Evaluation
Source: Healthcare (Basel). 2025 Feb 3;13(3):311. doi: 10.3390/healthcare13030311 (PMC11817868; doi:10.3390/healthcare13030311)
Supplement: Supplementary file 1 [file healthcare-13-00311-s001.zip › Supplementary Table 3 - Participant Themes and Quotes re Phase 1.pdf]

Supplementary Table S3: Developmental Evaluation Themes and Quotes from Participants Regarding their Co-design Experience during Phase 1

Themes	*Participants*	*Quotes*
**Understanding Care Setting Context and Issues**	PFA	“We understand some common issues across many areas of care, but not details like the data”; “important to know this data for area we will be working in”
	Staff/Care Provider	“Discussions concerning the [care settings] specific patient data was enlightening”; “an opportunity for units to build their capacity in using data more appropriately for quality improvement”.
**Interpreting Data – “making common sense of data”**	PFA	Discussion leads participants to data interpretation – i.e. what experiences are generally good, and also a “better understanding of possible areas needing improvement”; “Surprised to see rather low scores for important patient experiences.”
	Staff/Care Provider	“Every so often, it’s good to discuss QI and the process – we take it for granted sometimes or think that QI isn’t going to change anything anyway”.
**Framing relevant experience questions**	PFA	“Framing questions is an important role – would have been hard to know where to start if it wasn’t for all of us, as a team, interpreting the existing data for the unit first. Asking the right questions, in the right way, will make a huge difference with how patients will respond”.<br>“Exciting to be part of the actual design of the survey we will use with patients when we talk with them”;<br>“I began to wonder during the survey [development] – what does this matter? Whose focus was it? Did the questions matter to the patients?”<br>“I enjoyed the collaborative work to create the tools… could identify the start, middle and finish”
	Staff/Care Provider	“Challenging at first to spend the time working with everyone to develop the survey, but well worth making sure everyone agrees on what we ask and how – makes a big difference”;<br>“…respect what others suggest for what’s important to ask a person”

**Creating reasonable experience measurement surveys**	PFA	"…seeing the survey we all contributed to creating was amazing!"; "Not sure what the staff wanted in the survey or from the survey – what did they expect?'.<br>"… wasn't sure how we were all going to be involved in the survey development but it seemed to work out very well – everyone was in agreement with questions needed and why";<br>"Co-design needs to be in place, otherwise the survey will become too nurse or healthcare related; patient advisors must be in attendance to develop the survey".
	Staff/ Care Provider	"We all seemed to appreciate being patient with the process of developing surveys that would be reasonable for patients and care providers to respond to"; "…love the idea of having some similar questions for both patient and staff surveys … will be interesting to compare differences and similarities";<br>"Impressed with the process of developing the surveys – worked well".
**Exploring experiences with COVID**	PFA	"Doing this work during COVID presented us with additional challenges – working together virtually but also deciding on questions as part of homework";<br>"More specific questions on COVID needed to be discussed – comparing during COVID to before COVID"
	Staff/Care Provider	"not always able to have staff/care provider complement at meetings to decide on survey questions, especially related to impact of COVID, which are important – not sure what to ask when we are right in the midst of COVID impacted in many ways – almost a study in itself…"
