# Supplementary material for: Exploring a Co-Designed Approach for Healthcare Quality Improvement—Learning Through Developmental Evaluation
Source: Healthcare (Basel). 2025 Feb 3;13(3):311. doi: 10.3390/healthcare13030311 (PMC11817868; doi:10.3390/healthcare13030311)
Supplement: Supplementary file 1 [file healthcare-13-00311-s001.zip › Supplementary Table 4 - Participant Themes and Quotes re Phase 2.pdf]

Supplementary Table S4: Developmental Evaluation Themes and Quotes from Participants Regarding their Co-design Experience during Phase 2

Themes	*Participants*	*Quotes*
**Feeling Supported in care settings to gather data**	PFA	having "all the support needed – initially wondering if staff would be on board with survey but found staff to be receptive and welcoming… became comfortable and removed all assumptions about staff"; "As a peer of those patient/families I am gathering experience data from, I felt they trusted me and could tell me the truth about their care and experiences"
	Staff/Care Provider	"Support for pilot work was huge! It came from everyone on our unit and leadership as well"; "staff on unit were receptive to having PFAs talking with patients and gathering survey data"; "[Pilot] facilitators were always there and available for support or bouncing off feelings and ideas as data was being gathered and analyzed in real time for the site."; "interesting to see PFAs gathering experience data from other patients/families on our unit."
**Gathering Real-time Data – what matters to patients**	PFA	Used the lens of "what matters to patients" when gathering patient/family responses and comments; "Talking with patients/families took a lot of time, time not often acknowledged by our team, particularly the care providers – don't think they knew exactly what we did or what was involved as we talked with patients/families about their experiences".
	Staff/ Care Provider	"It's amazing how gathering patient experiences in real-time by the Advisors meant so much to us, as we are never sure we get 'what matters to patients' when we are talking with them"; "having real-time patient experience data to guide our QI work is exactly what we need – waiting for other data from AHS takes too long".
**Analyzing and Interpreting Experience Data in real time**	PFA	"…exciting to be part of the data analysis and interpretation discussion… never really involved in that before"; "… seeing real-time data and what it means for the unit [care setting] is a learning process for anyone like me, as an Advisor"
	Staff/Care Provider	"Really loved having real-time pre-post QI patient data to discuss and interpret to actually inform our QI activities, and how successful we are at making changes"; "having our own unit data to interpret is actually empowering".
