# Supplementary material for: Exploring a Co-Designed Approach for Healthcare Quality Improvement—Learning Through Developmental Evaluation
Source: Healthcare (Basel). 2025 Feb 3;13(3):311. doi: 10.3390/healthcare13030311 (PMC11817868; doi:10.3390/healthcare13030311)
Supplement: Supplementary file 1 [file healthcare-13-00311-s001.zip › Supplementary Table 5 - Participant Themes and Quotes re Phase 3.pdf]

Supplementary Table S5: Developmental Evaluation Themes and Quotes from Participants Regarding their Co-design Experience during Phase 3

Themes	*Participants*	*Quotes*
**Understanding QI**	PFA	Discussion leads participants to a "better understanding of possible areas needing improvement";<br>"need more clarity around QI process"
	Staff/Care Provider	"Every so often, it's good to discuss QI and the process – we take it for granted sometimes or think that QI isn't going to change anything anyway".
**Having a Flexible Approach**	PFA	"…appreciated the flexible process";<br>"Would have appreciated more structure at times around the discussion of which QI intervention or strategy to start with and why".
	Staff/ Care Provider	"We all seemed to appreciate the flexible approach to developing the QI plan ….didn't really know where to start and needed to muddle our way through sometime"
**Engaging in QI Planning Decisions**	PFA	"This phase of the pilot was very vital as it gave me more insight into what to expect and what I was doing up to this point with gathering experiences of patients/families – needed to see how the data was going to be used."<br>"…important to feeling comfortable being part of the group discussion in planning Qi, including following up to see how effective it is"
	Staff/Care Provider	"…appreciated the organization and planning that went into ensuring discussions would be focused and result in an actual agreed-on plan".
**Comparing data for Pre and Post QI**	PFA	"…exciting to be part of the data analysis and interpretation discussion… never really involved in that before"; "… seeing real-time data and what it means for the unit [care setting] is a learning process for anyone like me, as an Advisor"
	Staff/Care Provider	"Really loved having real-time pre-post QI patient data to discuss and interpret to actually inform our QI activities, and how successful we are at making changes"; "having our own unit data to interpret is actually empowering".
**Making a Difference**	PFA	They needed to know and feel they were "part of the co-design team", "making a difference" as "patients/families asking patients/families questions", and "having the real-time patient voice heard" to support change;<br>"…valued high level partnership"<br>"Great discussion – everyone involved";

		"It's hard to know what to always say – staff know what they want. How do we participate to add interpretation?"; "I was impressed by the enthusiasm and energy of the [home care] staff involved in the pilot… they were very engaged in wanting to improve their program services and the reputation of the home care program in their community, and this was all done even though they were very busy in trying to complete their daily work. When I commented during discussions they seemed to take my comments seriously and almost always asked questions for more clarity and to understand my comments."
	Staff/Care Provider	"The pre-post measurement of patient and staff experiences related to our QI initiative made me feel that we were making a difference for patients and for ourselves"; "…can see we are making a difference on one issue in this pilot, but hope we can keep this process going to ensure we can make a difference on other issues too"; "Good to hear what patients and family members think – don't always get the chance to have discussions about the issues on our unit"; "…respect what others have to say but not always easy to change"
