# Supplementary material for: Exploring a Co-Designed Approach for Healthcare Quality Improvement—Learning Through Developmental Evaluation
Source: Healthcare (Basel). 2025 Feb 3;13(3):311. doi: 10.3390/healthcare13030311 (PMC11817868; doi:10.3390/healthcare13030311)
Supplement: Supplementary file 1 [file healthcare-13-00311-s001.zip › Supplementary Table 6 - Participant Themes and Quotes re Phase 4.pdf]

Supplementary Table S6: Developmental Evaluation Themes and Quotes from Participants Regarding their Co-design Experience during Phase 4

Themes	*Participants*	*Quotes*
**Communication Throughout**	PFA	"Communication is key!"<br>As QI happened…"gained increased communication and contact with staff; increased education";<br>"…appreciated communication with group [i.e. steering committee] during touch points and from facilitators [of pilot]"; "meetings went well and we were listened to";<br>"Conference calls re survey worked well, but would appreciate more face-to-face discussion maybe halfway through pilot"; or "maybe a little more direct contact after committee meetings and during the surveying process (just as encouragement, problem solving on how to best reach clients), …where our input was appropriate and where it wasn't as I felt that I shouldn't participate to much in the analysis of information or the change action development… I did not reach out either, so as scheduled short discussion via teleconference, not email, may have been better".
	Staff/Care Provider	"communication from pilot coordinators throughout the pilot work kept us moving forward, as did the open less formal communication with the PFAs";<br>"need to be open to change process and co-design approach – not easy…"
**Valued Experiences**	PFA	"great experience"; "felt valued", "part of change process"; "really enjoyed this opportunity" and "perfect involvement"; "valued opportunity to be part of this process and QI pilot"; "I was so glad I did this – I am not an island, we are not an island"; Gone from total intimidation to being part of a team";<br>Seeing "everyone coming together to co-design – process offers a way to learn and see AHS cares and makes changes";<br>"that we can come together, we can collaborate and co-design a process for all the best intentions and do good for people – I can see that happen".
	Staff/Care Provider	"…found value in the co-design process with the Advisors… enlightened and strengthened the credibility around QI which really is all about improving patient experiences";

**Benefits**	PFA	"Real-time patient voice heard in co-designed QI";<br>"realizing patient needs can be given to unit with results 'now' particularly as patients were willing to share their experiences with Advisors – less likely with staff";<br>"…see co-design as putting PFCC [Patient-and-Family-Centred-Care] into action";<br>"it [co-design] is imperative – advisors have more confidence to give input, become familiar with issues and questions needing to be asked'.
	Staff/Care Provider	"…co-designing for QI is of benefit – works well with working group of staff and advisors – a coordinated team effort… structure is then easier to provide and follow";<br>"co-design enhances the unit capacity to do good work in gathering patient experiences in real-time and in having staff better understand patient voice in QI";<br>'shared accountability across all involved – learn together and work together to make a difference"
**Limitations/ Challenges**	PFA	"understanding entire process"; "everyone needs to understand it before doing it";<br>"AHS needs to have training in place for Advisors to do this kind of work… should be able to have ready advisors interested in being involved and maybe have back-ups at each unit as well – at least two advisors to do the work on a unit…";<br>"gathering real-time patient experiences using iPads was sometimes challenging if internet was not working…need paper or other options as backup";<br>"on some units, the challenge and concern was that staff – staff not consistent – disorganized … didn't remember or know we were there to talk with patients about their experiences… had to be reminded of purpose/reason for being there".
	Staff/ Care Provider	Looking at "active coordination" (i.e. "whose job is this?") and "time involvement as part of their regular work time"; "capacity building at the unit level would be needed to ensure everyone understands the co-design process and what is involved in working with Advisors directly on the unit";<br>"..need to have access to trained and available Advisors on a regular basis to do good QI";<br>"not all units can afford to have iPads to gather and analyze real-time patient experiences – this is a cost item"
**Co-design needed and feasible**	PFA	"…would like to see more co-designed QI initiatives across AHS, and wonder if it can be used in other work as well?"

		"Co-designing with PFAs is needed"; "good to have at AHS";<br>"pilot demonstrated how feasible this is";<br>"Cost is low but the value is huge";<br>"Appreciate the co-design – offers more thought, ideas, concepts; bounce ides off each other; input from relevant persons especially the family and patients".
	Staff/Care Provider	"want to see this process continue" and "across all areas within AHS";<br>"Process and learnings need to be shared broadly – want to see this approach continue"; "need ongoing workshops or training for staff across AHS";<br>"Designing this [co-design process] is feasible and sustainable with the right staff champion who can get people mobilized".
**Suggestions to keep Co-design Feasible and Sustainable**	PFA	**Early relationship building – trust**: "a bit of information time before the meetings was very helpful in making me feel comfortable and part of the team even if I wasn't part of the AHS Home Care program. The food, informal discussion and the time that was taken for individual introductions was very helpful";<br>**Orientation**: "this was essential to the overall success of the pilot work – it established a good meeting ground"<br>**Clear roles/responsibilities**: "I sometimes wondered about my role as I think I was there to provide the information back from the surveys or reflect on how the survey questions would be perceived by the clients and I think the staff was open to those thoughts"; "learned that there is a switch or change in hats when surveying as an advisor – maybe need more discussion on role".<br>**Collaboration:** "yes, it is feasible but depends on participants at the table and if same goals is agreed upon"; "Collaboration is integral between the designers of the project – staff, advisors – can make or break the work. I was so glad I did this!"; "staff were cooperative and provided support throughout the process"; Enjoyed collaborative work to create the tools – could identify the start, middle, and finish; could identify as part of the co-design team";<br>**Communication throughout:** "communication is key"; "communication was really good – things were framed positively which is a skill – I really felt the appreciation for my input, kept me going, kept my interest as a member of the team"; "Advisors needed to be kept informed of process by team members, especially by staff on unit as to when they were cleared to talk with

		patients"; "needed clear understanding and discussion of time involvement for different tasks – some took a tot longer than expected";<br>**More Advisors needed for backup**: "suggest to have more volunteers ready to become trained to so this kind of work"<br>**Additional preparation/training:** "if not used to speaking with people, may need more orientation/training re process"**;** " may need more background on how QI initiatives work"; "Some preliminary discussion on how to complete the surveys, probing to get answers, making individuals feel comfortable may make the process for volunteers easier. Insight into the survey questions by patient and family advisors is a very positive part of the process as we work together with staff/others".<br>**Ownership of a different process:** "depends on who is at the table – everyone needs to be open to change and owning it"; acquired appreciation for how much work is needed in making changes by staff and looking at this through a new lens of patient care and how care impacts patients" – gave me a whole new perspective of other side of the fence"; "I think this is different than getting opinions of 'lived experiences' from a few advisors on particular areas, as opinions expand beyond a few advisors and volunteers to gathering the opinions of many service users and the public"<br>**Facilitation – guidance and support:** "Facilitators have to be strong to guide the team – felt in our case, they were amazing"; "Facilitators kept us all on track including with the group's focus – co-design was a positive nugget that adds and becomes a good thing. If you lose the focus of why and who it is this is all for, then everything is broken"; "felt very assured, safe and comfortable with the facilitators – we were encouraged to be engaged as part of the co-design team, share our ideas, and were listened to, respected, and appreciated for our contributions".
	Staff/ Care Provider	**Early relationship building** – trust: "Needed ice breakers to help us open up to the idea of working together with patient advisors – a first for me!";<br>**Orientation:** "an essential part for starting us off on the right path for co-design efforts, and keeping us interested"<br>**Clear roles/responsibilities:** 'Wasn't sure about how things would work out for PFAs and staff working

	together on this project on the unit - and wasn't clear initially about their role in deciding on survey questions, or even the data and how it informed our QI intervention"; **Collaboration:** "great to work with advisors and get results 'now' in real time to help with making changes"; **Communication throughout:** "communication was key throughout for ensuring everyone was understanding what was happening and when"; 'easy to get lost if were are not kept in the loop on each step of the initiative"; **Additional Staff/Care provider preparation for Advisors arrival:** "essential to have mini-prep talks with staff to prepare them about the QI work and how advisors will be involved"; **Ownership of different process:** "not always clear what joint expectations are with this type of initiative – what do advisors get from this, what benefit?"; "co-designing this work made so much sense as we worked our way through things and saw the results" **Facilitation – guidance and support:** "it was clear from the outset that the facilitators guiding us through the orientation and work knew what they were doing – we began to trust the process as they provided us with guidance and support to do the work and follow the evidence through our QI process";
